# Contemporary Management of Vesico-Urethral Anastomotic Stenosis After Radical Prostatectomy

**DOI:** 10.3389/fsurg.2020.587271

**Published:** 2020-11-26

**Authors:** Clemens M. Rosenbaum, Margit Fisch, Malte W. Vetterlein

**Affiliations:** ^1^Department of Urology, Asklepios Klinik Barmbek, Hamburg, Germany; ^2^Department of Urology, University Medical Center Hamburg-Eppendorf, Hamburg, Germany

**Keywords:** prostatic neoplasms, urethral obstruction, transurethral resection, transurethral incision, urethral reconstruction

## Abstract

Vesico-urethral anastomotic stenosis is a well-known sequela after radical prostatectomy for prostate cancer and has significant impact on quality of life. This review aims to summarize contemporary therapeutical approaches and to give an overview of the available evidence regarding endoscopic interventions and open reconstruction. Initial treatment may include dilation, incision or transurethral resection. In treatment-refractory stenoses, open reconstruction via an abdominal (retropubic), transperineal or combined abdominoperineal approach is a viable option with high success rates. All of the open surgical procedures are generally accompanied by a high risk of developing *de novo* incontinence and patients may need further interventions. In such cases, subsequent artificial urinary sphincter implantation is the most common treatment option with the best available evidence.

## Introduction

Prostate cancer (PCa) represents the most frequent, solid malignant tumor among men in the Western hemisphere (1) and more than 80% of patients with localized PCa opt for definite treatment (2). Besides radiotherapy, one of the most common treatment option is radical prostatectomy (RP). Urinary incontinence and erectile dysfunction represent well-known and well-described treatment-related adverse events (3). Another common mid- to long-term complication after PCa treatment is bladder outlet obstruction (BOO) (4, 5). Given the relatively high overall and cancer-specific survival at 10 years (90% and 99%, respectively) (6), there is a relevant proportion of patients at risk of such long-term sequelae.

We believe it is important to emphasize that the term “urethral stricture” should be exclusively restricted to those parts of the urethra, which are surrounded by corpus spongiosum. This excludes the prostatic urethra at the outset (7). Moreover, it seems inevitable to us to distinguish between a bladder neck contracture (BNC) after surgical procedures for benign prostatic hyperplasia and VUAS after RP (7). It is a known fact, that etiology, anatomy, recurrence rates, and functional outcomes differ significantly between BNC and VUAS (8). BOO after PCa treatment includes radiation-induced bulbomembranous urethral stricture (9) as well as VUAS after RP (10). The following comprehensive narrative review aims to provide a contemporary summary of the epidemiology, etiology, preoperative evaluation, and treatment strategies for VUAS.

## Epidemiology

Evaluating the existing literature on VUAS, it is of utmost importance to keep in mind that VUAS is mainly defined as a condition resulting in a surgical procedure based on a patient's complaint. To the very best of our knowledge, there are no prospective studies available, which analyzed urethral patency after RP by any standardized diagnostic procedure. Thus, in most studies, any surgical procedure is considered as the diagnosis of VUAS. This may translate into a certain underestimation of the true VUAS incidence. In 2007, an analysis of the Cancer of the Prostate Strategic Urologic Research Endeavor (CaPSURE) database provided a detailed insight into epidemiology of BOO related to prior PCa therapy. Overall obstruction rate among all treatment modalities was 5.2% at a median follow-up of 2.7 years. Highest prevalence of BOO occurred in patients after RP (8.4%) (11). Remarkably, BOO rates in patients treated with RP and adjuvant or salvage radiotherapy were lower (2.7%).

Generally, it appears that VUAS incidence has declined over the years. Table 1 summarizes the evidence on VUAS incidence over the last two decades (5, 11–18). Of note, VUAS after robot-assisted laparoscopic RP seem to be less common as compared to open RP (~1.3 vs. 3.6%, respectively) (5, 14, 17, 19). These data suggest that not only the refinement of surgical techniques over time, but also (robotic or open) RP in experienced surgical hands and in high-volume centers will result in lower VUAS rates. Notably, VUAS rates in men who had to undergo salvage RP after failed radiotherapy is significantly higher (22–40%). However, this evidence originates from small case series (20, 21). Beyond VUAS, salvage therapies come along with a much higher risk of urinary incontinence, rectal injury and urorectal fistulae (22).

**Table 1:** Incidence of vesico-urethral anastomotic stenosis after radical prostatectomy as reported in the last two decades.

**First author**	**Year of publication**	**Number of patients**	**Study design**	**Follow-up**	**VUAS incidence**
**Open retropubic prostatectomy**					
Borboroglu et al. (12)	2000	467	Single-center	mean: 54 months	11%
Hu et al. (13)	2003	2,292	Multicenter	N/A	26%
Elliott et al. (11)	2007	3,310	Multicenter	median: 32 months	8.4%
Erickson et al. (14)	2009	4,132	Single-center	median: 44 months	2.5%
Carlsson et al. (15)	2010	458	Single-center	median: 30 months	4.5%
Gillitzer et al. (16)	2010	2,052	Single-center	median: 52 months	5.5%
Breyer et al. (17)	2010	695	Single-center	median: N/A; ≥ 12 months in all patients	2.6%
Modig et al. (5)	2019	942	Multicenter	mean: 24 months	3.6%
**Laparoscopic robot-assisted prostatectomy**					
Carlsson et al. (15)	2010	1,253	Single-center	median: 19 months	0.2%
Breyer et al. (17)	2010	293	Single-center	median: N/A; ≥ 12 months in all patients	1.4%
Parihar et al. (18)	2014	930	Single-center	mean: 23 months	1.6%

*VUAS, vesico-urethral anastomotic stenosis*.

## Etiology

Preoperative known measurable risk factors for the development of VUAS are obesity, smoking, diabetes, and hypertension (12). These factors may result in decreased microvasculature, possibly leading to prolonged healing of the vesico-urethral anastomosis. Transurethral resection of the prostate prior to RP and a large prostatic volume have been proven as risk factors of VUAS as well (12, 23). Intraoperative risk factors for VUAS are extensive blood loss, mismatch, and tension on the anastomosis (12, 24) whereas running sutures of the anastomosis as well as robot-assisted compared to open procedures are supposed to lower the risk (5, 17, 25). In general, VUAS occurs within the first 6 months after surgery. The incidence of VUAS significantly decreases 2 years after RP (11).

## Diagnostic Workup

Preoperative workup of VUAS should always include the medical history, previous procedures, and an evaluation of length and location of the stenosis (26). Clinical presentation usually includes obstructive symptoms such as a weak stream, hesitancy, and post-void residual urine. Moreover, patients who underwent adjuvant or salvage radiotherapy after RP often present with urgency and frequency symptoms with or without urinary incontinence.

If there is any surgical treatment planned, a prostate-specific antigen test should be performed to rule out PCa recurrence. Diagnosis of recurrent PCa would lead to different treatment strategies. Uroflowmetry and post-void residual urine measurement should objectify obstructive symptoms.

Radiologic investigation represents another important part of the diagnostic workflow. Combined retrograde urethrography (RUG) and voiding cystourethrography (VCUG) gives valuable information about the status of the anterior and posterior urethra. Moreover, combination of RUG and VCUG reveals a “funneled” VUAS (27). This “funneled” VUAS may impair the exact identification of VUAS location and length. As the anastomosis during RP is performed by connecting bladder neck and membranous urethra, the funneled area can be part of the VUAS. This may result in involving the membranous urethra and therefore the external urethral sphincter. Therefore, another integral part of the diagnostic workflow is a cystoscopy. Stenotic involvement of the external sphincter can be evaluated more precisely compared to isolated radiographic evaluation and urethral diameter can be adequately assessed. Given that incontinence rates are twice as high in patients with a VUAS compared to those without VUAS (5), pad test and evaluation of patient-reported outcome measurements (PROMs) should be performed prior to any surgical intervention to assess the baseline continence status.

## Treatment

### Endoscopic Procedures

**Figure 1:**
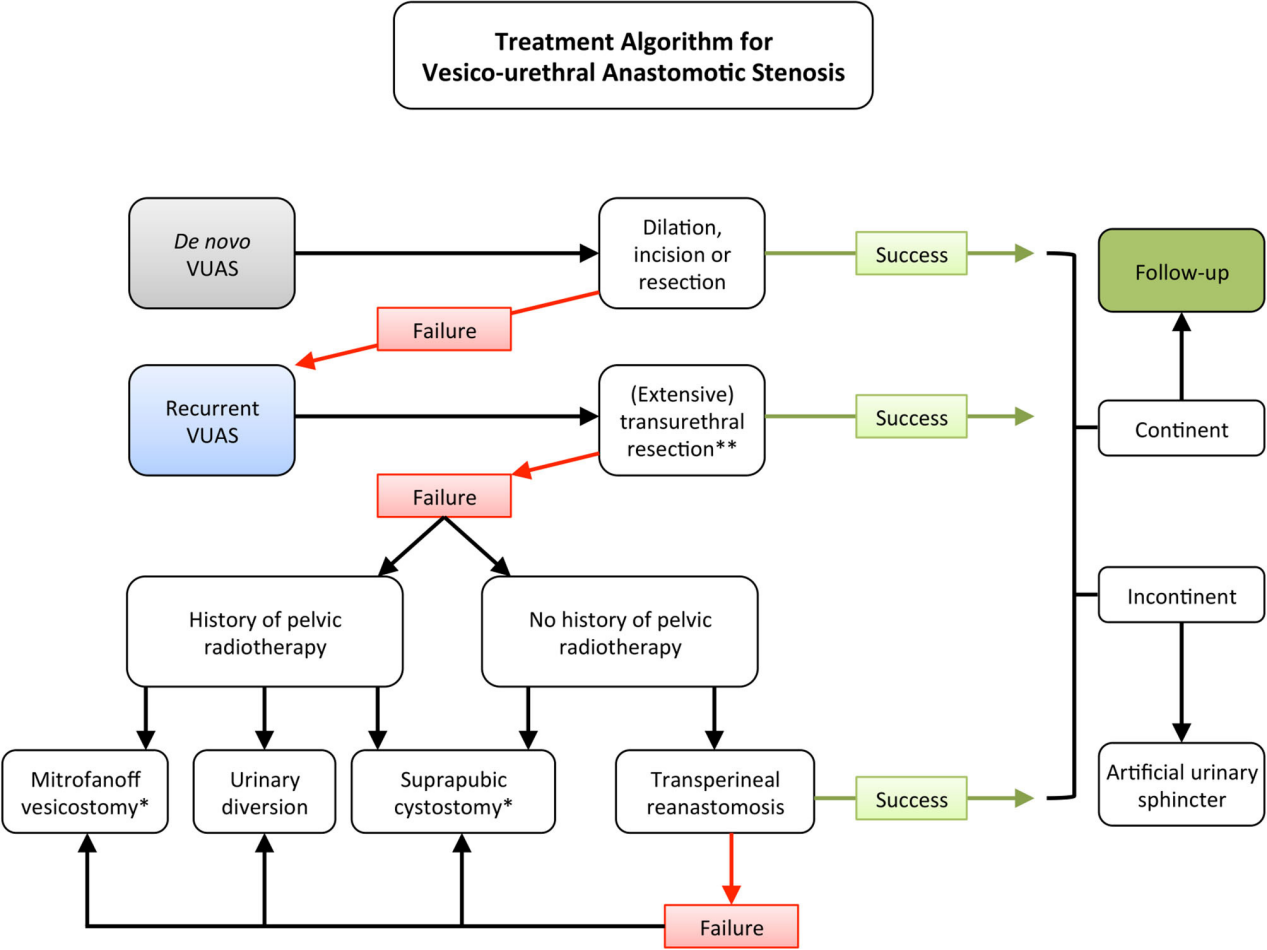
Proposal of a treatment algorithm for vesico-urethral anastomotic stenosis (VUAS) following radical prostatectomy (*Hamburg VUAS Algorithm*). * Simultaneous permanent urethral ligation in case of urinary incontinence. ** Not more than three times.

Treatment algorithms for VUAS should usually commence with endoscopic therapy (Figure 1). Whereas, the European Association of Urology (EAU) guideline on urological trauma suggests dilation or transurethral incision (28), the American Urological Association (AUA) recommends a treatment decision at the surgeon's discretion (dilation, incision, or resection) (29). The most comprehensive recommendation regarding the sequential treatment of patients with VUAS is provided by a collaboration of the Société Internationale D'Urologie (SIU) and the International Consultation on Urological Diseases (ICUD) (30). A priori, patients are stratified according to continence status. In incontinent patients, the guidelines differentiate between a completely obliterated urethra with the recommendation to perform suprapubic cystostomy followed by open reconstruction as a first line strategy. In incontinent patient with residual urethral patency, transurethral incision with or without continuous intermittent catheterization is recommended. For continent patients, the SIU/ICUD guideline recommends dilation or incision as a first line therapy (30). It is important to mention that all of such recommendations are based on data with low level of evidence.

If the membranous urethra is involved, most authors favor dilation as a first line therapy (27), which may already lead to reasonable success rates (31).

Success rates after primary incision or resection range between 37 and 69%. This rate may increase up to 91% after numerous sequential surgical procedures (8, 10, 32). There are only two publications to exclusively report on endoscopic treatment of VUAS and most of the published series do not distinguish between a BNC after surgery for benign prostatic hyperplasia and VUAS after RP. Table 2 summarizes the results from those two studies (10, 32).

**Table 2:** Endoscopic treatment of vesico-urethral anastomotic stenosis after radical prostatectomy.

**First author**	**Overall treatment**	**Treatment success in patients**	**Treatment success in patients**
	**success; *n* (%)**	**with no previous endoscopic treatment; *n* (%)**	**with ≥ 1 previous endoscopic treatment; *n* (%)**
**Holmium laser incision**			
LaBossiere et al. (32)	89/162 (55%)	48/70 (69%)	41/92 (45%)
Pfalzgraf et al. (10)	N/A	N/A	N/A
**Cold knife incision**			
LaBossiere et al. (32)	5/15 (33%)	2/8 (25%)	3/7 (43%)
Pfalzgraf et al. (10)	19/36 (53%)	N/A	N/A
**Transurethral resection**			
LaBossiere et al. (32)	26/64 (41%)	14/36 (39%)	12/28 (43%)
Pfalzgraf et al. (10)	25/67 (37%)	N/A	N/A
**Dilation**			
LaBossiere et al. (32)	6/46 (13%)	0/17 (0%)	6/29 (21%)
Pfalzgraf et al. (10)	N/A	N/A	N/A

Transurethral incision of the VUAS is usually performed at two sites. It should be emphasized that incision at the six o'clock position should be avoided. After RP, there is usually only a thin tissue plane between the vesico-urethral anastomosis and the rectum. Therefore, incision at this location would be prone to fistula formation or rectal injury (24). There is no high-level evidence on whether the incision should be performed by (hot or cold) knife or by laser (holmium or thulium). However, there is one publication suggesting a certain superiority of the holmium laser incision over cold knife incision (32). Injection of triamcinolone or mitomycin in addition to incision for recurrent VUAS has been described with success rates of 83–89% (33, 34). In this context, potential serious adverse events such as osteitis pubis, bladder necrosis, or rectourethral fistula with eventual need of cystectomy and supravesical diversion should be kept in mind and the risks and benefits should be adequately weighed (35). Another effort to treat recurrent VUAS has been made by using the UroLume stent (36). However, long-term follow-up has lowered initial expectations (37).

As mentioned above, the SIU/ICUD guidelines base treatment recommendations on a patient's continence status (30). The association of VUAS with incontinence is not uncommon (5). One possible explanation is that extensive fibrosis may involve the external sphincter, described as funneling by some authors (27). However, data about incontinence after endoscopic surgery for VUAS are rare. Pfalzgraf et al. have reported on postoperative *de novo* incontinence after endoscopic approaches in almost one third of patients. Incision resulted in higher incontinence rates as compared to resection (31 vs. 12%, respectively), whereas no difference was observed for previously irradiated vs. non-irradiated or primary vs. repeatedly treated patients (10).

### Open Surgical Reconstruction

All endoscopic therapies inherit the risk of recurrence. Therefore, there is a non-negligible number of patients with recurrent VUAS. In those patients, transurethral therapy should not be continued and open surgical reconstruction should be discussed with the patient (Figure 1). We generally opt for open reconstruction in case of treatment failure after three previously failed transurethral procedures. Treatment choices should be patient-centered. Therefore, bladder drainage by permanent catheterization (transurethral or suprapubic) may be one option, especially for frail and very old patients. However, in most cases it is worth considering an open reconstruction of the VUAS. In very complex situations, urinary diversion may be another option but should be regarded as a last resort.

Different approaches have been discussed for open reconstruction: the abdominal (retropubic), the (trans)perineal, and the combined abdominoperineal approach (27, 38–43). For all of these approaches, results have been generally satisfying. Table 3 gives an overview about the latest published evidence.

**Table 3:** Open surgical reconstruction of recurrent vesico-urethral anastomotic stenosis after radical prostatectomy.

**First author**	**Year**	**Number of patients**	**Follow-up**	**Treatment success**	**Comment**
**Abdominal approach**					
Pfalzgraf et al. (41)	2011	20	median: 59 months	60%	95% treatment success after secondary endoscopy
**Abdominoperineal approach**					
Theodoros et al. (38)	2000	6	mean: 24 months	83%	Simultaneous AUS implantation in all patients
					Simultaneous bladder augmentation in three patients
Elliott et al. (39)	2006	10	median: 24 months	70%	50% treatment success in irradiated patients
**Perineal approach**					
Reiss et al. (42)	2014	15	mean: 21 months	93%	100% treatment success after secondary endoscopy
Schüttfort et al. (43)	2017	23	mean: 45 months	87%	100% treatment success after secondary endoscopy

*AUS, artificial urinary sphincter*.

Lately, robotic reconstruction of VUAS has been added to the surgical armamentarium. In a recent case series of 12 patients including seven patients with BNC and five patients with VUAS, treatment success was 75%. *De novo* incontinence has been observed in 18% of patients (44).

When using the open retropubic approach, the bladder neck is accessed via an abdominal midline incision. VUAS scar tissue is excised and a reanastomosis is established similarly to primary vesico-urethral anastomosis during RP (41). Primary success rate can be as high as 60%. If further endoscopic therapies are performed for recurrences, overall success rate may raise up to 95% (41).

The transperineal approach inherits several advantages over the abdominal approach: First, adhesiolysis and surgical obstacles due to extensive scarred tissue in the previously operated field may be avoided. It can be difficult to identify surgical planes. Scar tissue resection can be challenging. Second, urethral mobilization to achieve a tension-free anastomosis can be difficult by the retropubic approach and may be facilitated by transperineal access (42). However, the transperineal approach can be very challenging (27). It seems mandatory that this procedure is performed in experienced centers.

For transperineal reanastomosis the patient is exposed in an exaggerated lithotomy position. A transperineal half-moon incision should be performed and the urethra should be dissected under digital-rectal examination. A complete exposition of the urethra and anastomotic area should be obtained. Scar tissue should be completely excised, beginning from the urethral lumen until healthy tissue is reached. A transurethral catheter allows for better orientation and identification of the distal end of the healthy urethra. Wide mobilization of urethra and bladder should be performed to guarantee a tension-free anastomosis (42). By some authors, a separation of the crura and sometimes even an inferior wedge pubectomy is recommended as the mobilization is generally done very far forward into the anterior triangle of the perineum (27). A dorsal spatulation of the anterior urethra should be performed and reanastomosis should be sutured by single knots under direct vision control. We propose inserting an 18 F transurethral catheter postoperatively (42). As recently shown, transperineal reanastomosis may result in success rates of up to 90% (43).

In previously irradiated patients, we would advise against performing a transperineal reanastomosis (Figure 1). One treatment option in those patients is to perform a continent vesicostomy (Mitrofanoff) with reasonable success rates (45). In patients with urinary incontinence, perineal ligation of the bladder neck should be performed simultaneously. However, in irradiated patients, bladder neck ligation can be challenging and success rates are lower compared to non-irradiated patients undergoing continent vesicostomy. Therefore, urinary diversion represents a reliable treatment option in this subgroup of patients (46).

Continence rates after open retropubic or robotic reanastomosis range between 18 and 31% in preoperatively continent patients (41, 44). After transperineal reanastomosis, almost all patients remain incontinent (43). It is therefore mandatory to counsel patients prior to reanastomosis about the possible necessity of a subsequent artificial urinary sphincter (AUS) implantation. A simultaneous reanastomosis and AUS placement is possible, but a two-staged procedure minimizes the risk of infection (31, 38). Additionally, staged procedures maintain the option of further endoscopic therapy in case of early VUAS recurrence. Ultimately, the stressed urethra is prone to revascularization. Urethral atrophy after cuff placement during AUS is therefore more unlikely. AUS placement should be performed 3–6 months after reanastomosis as completion of wound healing after this time period is very likely.

## Future Directions

In the light of 90% overall and 99% cancer-specific survival at 10 years of follow-up (6), there is a need to better classify VUAS severity and complexity. That said, treatment options ought to be tailored more precisely. A superior classification system could possibly be achieved by including magnetic resonance tomography (MRI) into the diagnostic workup. As of today, combined urethrography represents the standard diagnostic procedure. In some cases, a “funneled” VUAS can be detected (27). However, the relation to the external sphincter, the exact length of the stenosis, and severity of fibrosis surrounding the stenosis cannot be predicted precisely. As a standard diagnostic tool for prostate cancer, MRI could help to better understand the pathophysiology of VUAS and the aforementioned factors. Whereas, there is no relevant data on MRI in the context of VUAS, MRI compared to standard radiographic assessment showed a better predictive capacity regarding the length of stenosis in obliterated posterior urethral strictures (47, 48). Moreover, in traumatic bulbar urethral strictures, MRI appears more precise in anticipating the degree of spongiofibrosis, concomitant fistula, and stricture length compared to conventional diagnostic tools (49). A novel VUAS classification should—among others—possibly include stenosis grading and etiological aspects. Taken together, a VUAS classification system would have important implications for both patients and urologists to improve treatment choices and predict surgical outcomes. Furthermore, an accepted grading system could aid in choosing the optimal treatment option, as previous attempts to predict urethral patency after VUAS treatment have failed (10). As of now, the type of endoscopic treatment as well as the decision to move on to open reconstruction is mostly based on surgeon preference and institutional experience. There is a crucial need for prospective, multi-institutional randomized studies with a well-selected patient population.

## Conclusions

VUAS is one of the most common complications after RP. Fortunately, incidence has declined over the last decades and was reported at ~2% in recent series. VUAS usually occurs within the first 2 years after RP. Endoscopic treatment should usually be performed as a first line therapy, and most patients can be treated successfully. However, some patients develop recurrent VUAS. In those, reconstructive surgery should be considered. Reanastomosis, if performed by an abdominal, a perineal or a robotic-assisted laparoscopic approach, can result in high success rates. All types of VUAS therapy inherit the risk of *de novo* incontinence, which may be as high as 31 and 100% after endoscopic and open reconstruction, respectively. In these cases, AUS implantation can be regarded the most common treatment option with the best evidence available.

## Author Contributions

CR: conceptualization, methodology, investigation, writing - original draft, and project administration. MF: writing - review, editing, and supervision. MV: conceptualization, methodology, investigation, and writing - original draft. All authors contributed to the article and approved the submitted version.

## Conflict of Interest

The authors declare that the research was conducted in the absence of any commercial or financial relationships that could be construed as a potential conflict of interest.

## References

[1] Siegel RL Miller KD Jemal A Cancer statistics, 2020. CA Cancer J Clin. (2020) 70:7–30. 10.3322/caac.21590 31912902

[2] Gray PJ Lin CC Cooperberg MR Jemal A Efstathiou JA. Temporal trends and the impact of race, insurance, and socioeconomic status in the management of localized prostate cancer. Eur Urol. (2017) 71:729–37. 10.1016/j.eururo.2016.08.047 27597241

[3] Potosky AL Davis WW Hoffman RM Stanford JL Stephenson RA Penson DF Five-year outcomes after prostatectomy or radiotherapy for prostate cancer: the prostate cancer outcomes study. J Natl Cancer Inst. (2004) 96:1358–67. 10.1093/jnci/djh259 15367568

[4] Nam RK Cheung P Herschorn S Saskin R Su J Klotz LH Incidence of complications other than urinary incontinence or erectile dysfunction after radical prostatectomy or radiotherapy for prostate cancer: a population-based cohort study. Lancet Oncol. (2014) 15:223–31. 10.1016/S1470-2045(13)70606-5 24440474

[5] Modig KK Godtman RA Bjartell A Carlsson S Haglind E Hugosson J Vesicourethral anastomotic stenosis after open or robot-assisted laparoscopic retropubic prostatectomy-results from the laparoscopic prostatectomy robot open trial. Eur Urol Focus. (2019). 10.1016/j.euf.2019.10.012. [Epub ahead of print]. 31711932

[6] Hamdy FC Donovan JL Lane JA Mason M Metcalfe C Holding P 10-year outcomes after monitoring, surgery, or radiotherapy for localized prostate cancer. N Engl J Med. (2016) 375:1415–24. 10.1056/NEJMoa1606220 27626136

[7] Latini JM McAninch JW Brandes SB Chung JY Rosenstein D. SIU/ICUD consultation on urethral strictures: epidemiology, etiology, anatomy, and nomenclature of urethral stenoses, strictures, and pelvic fracture urethral disruption injuries. Urology. (2014) 83:S1–7. 10.1016/j.urology.2013.09.009 24210733

[8] Kranz J Reiss PC Salomon G Steffens J Fisch M Rosenbaum CM. Differences in recurrence rate and *de novo* incontinence after endoscopic treatment of vesicourethral stenosis and bladder neck stenosis. Front Surg. (2017) 4:44. 10.3389/fsurg.2017.00044 28848735 PMC5554361

[9] Vetterlein MW Kluth LA Zumstein V Meyer CP Ludwig TA Soave A. Buccal mucosal graft urethroplasty for radiation-induced urethral strictures: an evaluation using the extended Urethral Stricture Surgery Patient-Reported Outcome Measure (USS PROM). World J Urol. (2020) 38:2863–72. 10.1007/s00345-020-03102-5 32067075 PMC7644515

[10] Pfalzgraf D Worst T Kranz J Steffens J Salomon G Fisch M. Vesico-urethral anastomotic stenosis following radical prostatectomy: a multi-institutional outcome analysis with a focus on endoscopic approach, surgical sequence, and the impact of radiation therapy. World J Urol. (2020). 10.1007/s00345-020-03157-4. [Epub ahead of print]. 32236662

[11] Elliott SP Meng MV Elkin EP McAninch JW Duchane J Carroll PR. Incidence of urethral stricture after primary treatment for prostate cancer: data From CaPSURE. J Urol. (2007) 178:529–34. 10.1016/j.juro.2007.03.126 17570425

[12] Borboroglu PG Sands JP Roberts JL Amling CL. Risk factors for vesicourethral anastomotic stricture after radical prostatectomy. Urology. (2000) 56:96–100. 10.1016/s0090-4295(00)00556-2 10869633

[13] Hu JC Gold KF Pashos CL Mehta SS Litwin MS. Role of surgeon volume in radical prostatectomy outcomes. J Clin Oncol. (2003) 21:401–5. 10.1200/JCO.2003.05.169 12560426

[14] Erickson BA Meeks JJ Roehl KA Gonzalez CM Catalona WJ. Bladder neck contracture after retropubic radical prostatectomy: incidence and risk factors from a large single-surgeon experience. BJU Int. (2009) 104:1615–9. 10.1111/j.1464-410X.2009.08700.x 19583720 PMC3173809

[15] Carlsson S Nilsson AE Schumacher MC Jonsson MN Volz DS Steineck G. Surgery-related complications in 1253 robot-assisted and 485 open retropubic radical prostatectomies at the Karolinska University Hospital, Sweden. Urology. (2010) 75:1092–7. 10.1016/j.urology.2009.09.075 20022085

[16] Gillitzer R Thomas C Wiesner C Jones J Schmidt F Hampel C. Single center comparison of anastomotic strictures after radical perineal and radical retropubic prostatectomy. Urology. (2010) 76:417–22. 10.1016/j.urology.2009.10.009 19969328

[17] Breyer BN Davis CB Cowan JE Kane CJ Carroll PR. Incidence of bladder neck contracture after robot-assisted laparoscopic and open radical prostatectomy. BJU Int. (2010) 106:1734–8. 10.1111/j.1464-410X.2010.09333.x 20438567 PMC3565608

[18] Parihar JS Ha YS Kim IY. Bladder neck contracture-incidence and management following contemporary robot assisted radical prostatectomy technique. Prostate Int. (2014) 2:12–8. 10.12954/PI.13034 24693529 PMC3970984

[19] Webb DR Sethi K Gee K. An analysis of the causes of bladder neck contracture after open and robot-assisted laparoscopic radical prostatectomy. BJU Int. (2009) 103:957–63. 10.1111/j.1464-410X.2008.08278.x 19076148

[20] Ward JF Sebo TJ Blute ML Zincke H. Salvage surgery for radiorecurrent prostate cancer: contemporary outcomes. J Urol. (2005) 173:1156–60. 10.1097/01.ju.0000155534.54711.60 15758726

[21] Corcoran NM Godoy G Studd RC Casey RG Hurtado-Coll A Tyldesley S. Salvage prostatectomy post-definitive radiation therapy: The Vancouver experience. Can Urol Assoc J. (2013) 7:87–92. 10.5489/cuaj.11056 22277631 PMC3650760

[22] Heidenreich A Richter S Thuer D Pfister D. Prognostic parameters, complications, and oncologic and functional outcome of salvage radical prostatectomy for locally recurrent prostate cancer after 21st-century radiotherapy. Eur Urol. (2010) 57:437–43. 10.1016/j.eururo.2009.02.041 19303197

[23] Moul JW Mooneyhan RM Kao TC McLeod DG Cruess DF. Preoperative and operative factors to predict incontinence, impotence and stricture after radical prostatectomy. Prostate Cancer Prostatic Dis. (1998) 1:242–9. 10.1038/sj.pcan.4500248 12496883

[24] Browne BM Vanni AJ. Management of urethral stricture and bladder neck contracture following primary and salvage treatment of prostate cancer. Curr Urol Rep. (2017) 18:76. 10.1007/s11934-017-0729-0 28776126

[25] Albisinni S Aoun F Peltier A van Velthoven R. The single-knot running vesicourethral anastomosis after minimally invasive prostatectomy: review of the technique and its modifications, tips, and pitfalls. Prostate Cancer. (2016) 2016:1481727. 10.1155/2016/1481727 27340567 PMC4906212

[26] Vetterlein MW Rosenbaum CM Fisch M Surgical reconstruction of posterior urethral complications following prostate cancer treatments. In: Martins FE, Kulkarni SB, Köhler TS, editors. Textbook of Male Genitourethral Reconstruction. Cham: Springer International Publishing (2020). p. 303–17. 10.1007/978-3-030-21447-0_25

[27] Mundy AR Andrich DE. Posterior urethral complications of the treatment of prostate cancer. BJU Int. (2012) 110:304–25. 10.1111/j.1464-410X.2011.10864.x 22340079

[28] Summerton DJ Kitrey ND Lumen N Serafetinidis E Djakovic N European Association of U. EAU guidelines on iatrogenic trauma. Eur Urol. (2012) 62:628–39. 10.1016/j.eururo.2012.05.058 22717550

[29] Wessells H Angermeier KW Elliott S Gonzalez CM Kodama R Peterson AC. Male urethral stricture: American urological association guideline. J Urol. (2017) 197:182–90. 10.1016/j.juro.2016.07.087 27497791

[30] Herschorn S Elliott S Coburn M Wessells H Zinman L. SIU/ICUD consultation on urethral strictures: posterior urethral stenosis after treatment of prostate cancer. Urology. (2014) 83:S59–70. 10.1016/j.urology.2013.08.036 24361008

[31] Anger JT Raj GV Delvecchio FC Webster GD. Anastomotic contracture and incontinence after radical prostatectomy: a graded approach to management. J Urol. (2005) 173:1143–6. 10.1097/01.ju.0000155624.48337.a5 15758723

[32] LaBossiere JR Cheung D Rourke K. Endoscopic treatment of vesicourethral stenosis after radical prostatectomy: outcomes and predictors of success. J Urol. (2016) 195:1495–500. 10.1016/j.juro.2015.12.073 26719028

[33] Eltahawy E Gur U Virasoro R Schlossberg SM Jordan GH. Management of recurrent anastomotic stenosis following radical prostatectomy using holmium laser and steroid injection. BJU Int. (2008) 102:796–8. 10.1111/j.1464-410X.2008.07919.x 18671784

[34] Vanni AJ Zinman LN Buckley JC. Radial urethrotomy and intralesional mitomycin C for the management of recurrent bladder neck contractures. J Urol. (2011) 186:156–60. 10.1016/j.juro.2011.03.019 21575962

[35] Redshaw JD Broghammer JA Smith TG Voelzke BB Erickson BA McClung CD. Intralesional injection of mitomycin C at transurethral incision of bladder neck contracture may offer limited benefit: TURNS study group. J Urol. (2015) 193:587–92. 10.1016/j.juro.2014.08.104 25200807 PMC4307389

[36] Elliott DS Boone TB. Combined stent and artificial urinary sphincter for management of severe recurrent bladder neck contracture and stress incontinence after prostatectomy: a long-term evaluation. J Urol. (2001) 165:413–5. 10.1097/00005392-200102000-00014 11176385

[37] Anger J. Management of recalcitrant bladder neck contracture after radical prostatectomy for prostate cancer. UroLume stent. J Urol. (2011) 185:391–2. 10.1016/j.juro.2010.11.022 21168160

[38] Theodoros C Katsifotis C Stournaras P Moutzouris G Katsoulis A Floratos D. Abdomino-perineal repair of recurrent and complex bladder neck-prostatic urethra contractures. Eur Urol. (2000) 38:734–40. 10.1159/000020371 11111193

[39] Elliott SP McAninch JW Chi T Doyle SM Master VA. Management of severe urethral complications of prostate cancer therapy. J Urol. (2006) 176:2508–13. 10.1016/j.juro.2006.07.152 17085144

[40] Simonato A Gregori A Lissiani A Carmignani G. Two-stage transperineal management of posterior urethral strictures or bladder neck contractures associated with urinary incontinence after prostate surgery and endoscopic treatment failures. Eur Urol. (2007) 52:1499–504. 10.1016/j.eururo.2007.03.053 17418481

[41] Pfalzgraf D Beuke M Isbarn H Reiss CP Meyer-Moldenhauer WH Dahlem R. Open retropubic reanastomosis for highly recurrent and complex bladder neck stenosis. J Urol. (2011) 186:1944–7. 10.1016/j.juro.2011.07.040 21944115

[42] Reiss CP Pfalzgraf D Kluth LA Soave A Fisch M Dahlem R. Transperineal reanastomosis for the treatment for highly recurrent anastomotic strictures as a last option before urinary diversion. World J Urol. (2014) 32:1185–90. 10.1007/s00345-013-1180-6 24166286

[43] Schuettfort VM Dahlem R Kluth L Pfalzgraf D Rosenbaum C Ludwig T. Transperineal reanastomosis for treatment of highly recurrent anastomotic strictures after radical retropubic prostatectomy: extended follow-up. World J Urol. (2017) 35:1885–90. 10.1007/s00345-017-2067-8 28674908

[44] Kirshenbaum EJ Zhao LC Myers JB Elliott SP Vanni AJ Baradaran N. Patency and incontinence rates after robotic bladder neck reconstruction for vesicourethral anastomotic stenosis and recalcitrant bladder neck contractures: the trauma and urologic reconstructive network of surgeons experience. Urology. (2018) 118:227–33. 10.1016/j.urology.2018.05.007 29777787

[45] Spahn M Kocot A Loeser A Kneitz B Riedmiller H. Last resort in devastated bladder outlet: bladder neck closure and continent vesicostomy–long-term results and comparison of different techniques. Urology. (2010) 75:1185–92. 10.1016/j.urology.2009.11.070 20206979

[46] Riedmiller H Kocot A. The devastated bladder outlet: treatment options. Curr Opin Urol. (2015) 25:352–6. 10.1097/MOU.0000000000000185 26049880

[47] Oh MM Jin MH Sung DJ Yoon DK Kim JJ Moon du G Magnetic resonance urethrography to assess obliterative posterior urethral stricture: comparison to conventional retrograde urethrography with voiding cystourethrography. J Urol. (2010) 183:603–7. 10.1016/j.juro.2009.10.016 20018323

[48] Sung DJ Kim YH Cho SB Oh YW Lee NJ Kim JH. Obliterative urethral stricture: MR urethrography versus conventional retrograde urethrography with voiding cystourethrography. Radiology. (2006) 240:842–8. 10.1148/radiol.2403050590 16857977

[49] Horiguchi A Edo H Soga S Azuma R Shinchi M Ojima K. Magnetic resonance imaging findings of traumatic bulbar urethral stricture help estimate repair complexity. Urology. (2020) 135:146–53. 10.1016/j.urology.2019.09.036 31626854

